# Analysis of Deformation and Stresses of a Lightweight Floor System (LFS) under Thermal Action

**DOI:** 10.3390/ma14195727

**Published:** 2021-09-30

**Authors:** Jacek Karpiesiuk, Tadeusz Chyży

**Affiliations:** Faculty of Civil Engineering and Environmental Sciences, Bialystok University of Technology, Wiejska 45A, 15-351 Bialystok, Poland; t.chyzy@pb.edu.pl

**Keywords:** lightweight floor system, deformation, displacement, stress, thermal action, strain gauges

## Abstract

The lightweight floor system (LFS) with a heating coil is one of many types of radiant heating systems. It differs from most of the others, as it has a much higher thermal efficiency at low flow temperature. To verify whether adhesive mortars can safely connect the ceramic floor with the insulating substrate, the deformations and stresses values of all light system layers under thermal action should be checked and compared to their maximum strengths. For this purpose, an LFS test field was conducted using the strain gauges and digital measurement techniques, and floor displacements and deformations were determined. The results obtained from the tests were confirmed by finite element method calculations. It was also found that the stress of each floor component was much lower than their strength. This proves that the LFS with a heating coil, without metal lamellas, meets the safety regulation for use. The results of the analysis can be useful in the design of heated/cooled LFSs.

## 1. Introduction

Lightweight floor systems (LFSs) have been studied extensively, especially to determine comfort characteristics and heat flux density. In this article, research and analysis of an LFS with a heating coil was undertaken to evaluate mechanical strength. In the field of construction, the strength of various floor composites, as well as the temperature loads, are tested. In this article, we undertook mechanical strength analysis of a specific, individual technical solution under thermal action, which has only been studied thus far in the context of thermal performance and comfort. Positive results of the conducted research may stimulate the interest of scientists to subject this system to other analyses, as well as to use of this modern system more widely in the construction field.

The European standard [1] distinguishes seven types of radiant heaters, in which the heating medium is a liquid, most often water, flowing inside heating pipes of various diameters. These radiators can generally be called “heavy”, as in most of them, the heat transfer layer is a relatively heavy (“wet” or “dry”) concrete or anhydrite screed. The Norwegian standard [2] applies only to the lightweight floor system (LFS) in a horizontal arrangement without screeds, using heat-dissipating elements. They are most often metal foils or sheets, the so-called lamellas. The lack of screed in the LFS means that the system has low thermal inertia. The heat transfer work of the screed is taken over by thin-layer adhesives. The standard [2] can also be used when designing electric underfloor heating. According to the provisions of this standard, the remaining lightweight design solutions for radiant heating without conducting lamellas can be designed only through experimental tests. A typical structure of a lightweight heated floor, laid on a structural base, can be composed of various types of materials and the following layers:

- Thermal insulation with low thermal conductivity (wood-like boards, EPS, XPS, gypsum composites, and other similar materials);
- Heat-conducting layer (sheet, foil, lamellas);
- Heating pipes (coil) sunk in grooves of the thermal insulation;
- Artificial or natural flooring (ceramic tile, stone, wood, wood-like panels, PVC cladding, etc.) fixed with adhesives or laid loosely.

The unusual (not included in the standards) design of the lightweight heated floor, which is described in this article, is similar to the above arrangement, except for the heat-conducting layer, the lamellas.

**Figure 1:**
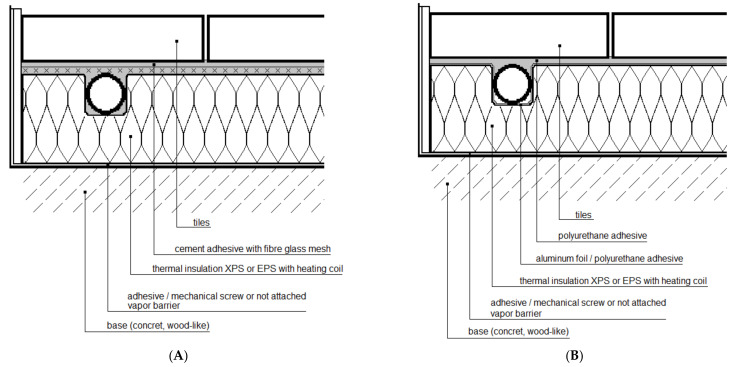
Cross-sections of a lightweight radiant heater (**A**) without a heat-dissipating layer (lamellas), and (**B**) with the heat-dissipating lamellas, namely aluminium foil.

**Figure 2:**
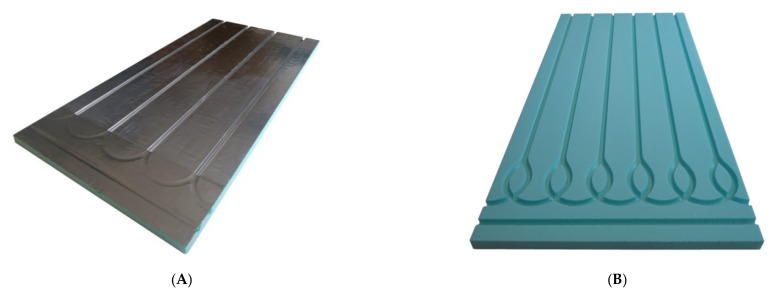
XPS insulation board (**A**) covered with aluminium lamella and (**B**) without lamella.

The experimental studies of the heat flux density and thermal inertia of lightweight, thin radiant heaters without screeds and with dry screeds at the Bialystok University of Technology, conducted by Zukowski and Karpiesiuk [3], Werner-Juszczuk [4], confirmed many advantages of a lightweight radiant heater without screeds. These studies have been defined as preliminary, with suggestions of carrying out future tests of the mechanical strength of this type of radiant heating structure. Figure 1 shows vertical cross-sections of radiant heaters of lightweight construction without screeds, adopted for similar tests of temperature distribution and thermal efficiency performed by Werner-Juszczuk [4] and Karpiesiuk and Chyzy [5]. In these tests, as the thermal insulation, expanded polystyrene (EPS) plates, with a compressive strength at 10% deformation of 200 kPa, and extruded polystyrene (XPS) plates, with a compressive strength of 300 kPa, were used (Figure 2). The most important conclusion of the research described in [5] was that a low-temperature, lightweight heated floor, both with and without lamellas, can be an efficient and user-friendly floor heater, provided that the distance between the heating pipes is up to 15 cm in the model with lamellas and up to 12.5 cm in the model without lamellas, with a coil spacing of 10 cm.

Taking into account the conclusions from the thermal tests, it was decided to perform strength tests on a lightweight heated floor without lamellas. There, the layers of ceramic flooring and XPS thermal insulation were joined with C2S1 deformable adhesive, reinforced with an E-type glass fibre mesh (GFRP). From the literature [6,7,8,9,10,11,12,13,14,15,16] it was not possible to obtain all the necessary strength and material parameter data of the layers in the adopted LFS without lamellas. Above all, the strength parameters of the adhesives used, connecting the floor with thermal insulation, were missing. To be sure that the LFS with a heating coil can safely be used in building partitions, many experimental tests have been performed on select materials. From these tests, the missing strength data and material parameters of the cement adhesive and its composite with GFRP mesh, such as Young’s modulus *E*, Poisson ratio *ν*, and thermal expansion coefficient *α*, were obtained and described in research performed by Karpiesiuk and Chyzy [17,18,19,20].

Experimentally obtained material and strength parameters allow for the development of a computational model for lightweight layered floors without screeds. Thus, it is possible to compare the results of numerical calculations with experimental research. For this purpose, experimental models were prepared to determine the deformations and displacements of LFS layers under the conditions of thermal action. Then, the results of experimental tests and computer calculations made with the use of the finite element method were compared and the stresses verified. The purpose of these tests and analyses, taking into account the maximum standard deflections, service loads, and thermal action of the floor, was to confirm or deny that the developed lightweight floor model without a screed layer and without aluminium lamellas, with a built-in coil, meets the strength and deformation conditions and can be used safely.

## 2. Materials and Experimental Methods

**Figure 3:**
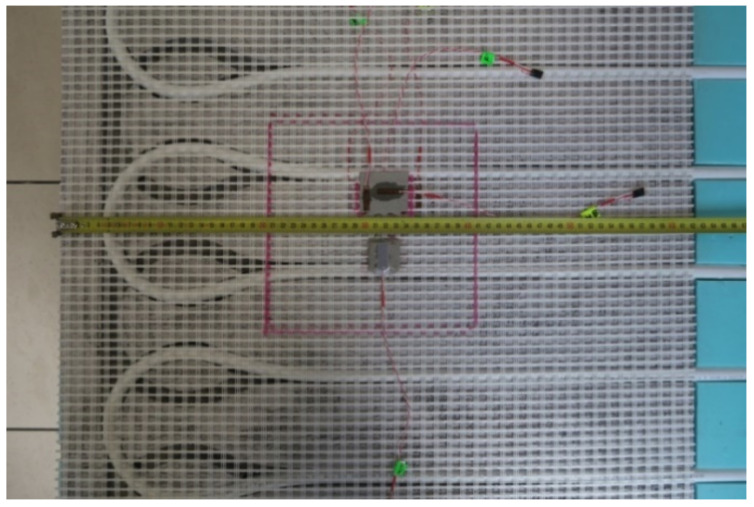
Attaching the strain gauges to C2S1 type adhesive, without lamellas.

It was decided experimentally to check the size of deformations and displacements in the LFS under the influence of temperature. For this purpose, a model of a lightweight heated floor with a heating coil was prepared without a heat-dissipating layer, namely the lamellas. The research was performed at the Bialystok University of Technology using the strain gauge technique and digital image correlation (DIC). The measuring model was 60 cm × 60 cm, and the floor was covered with 9 pieces of 20 cm × 20 cm ceramic tiles. Active strain gauges were placed at different levels of the adhesive layer, between the central tile and the XPS. Before embedding, the strain gauges were attached to the previously prepared adhesive substrates. Figure 3 shows the placement of the strain gauges mounted on the C2S1 adhesive.

**Figure 4:**
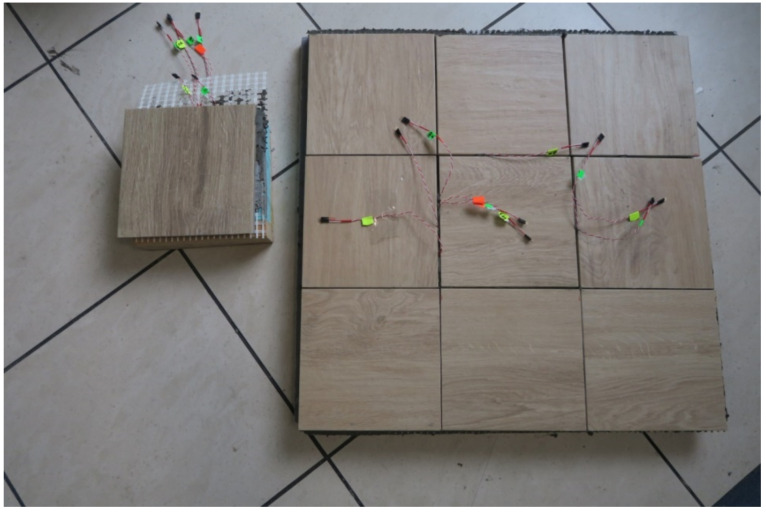
Research model with C2S1 adhesive mortar, reinforced by mesh, without lamellas.

The experimental model of the lightweight floor consisted of 40 mm XPS thermal insulation with grooves and a 16 mm diameter heating coil. The heating pipes were not fed with water, but with an Elektra UltraTec heating cable with unit power of 10 W/m, which is allowed by the Norwegian standard [2]. A 5 mm layer of C2S1 type adhesive mortar with 320 g/m^2^ fibreglass mesh embedded within it was placed on the insulating substrate. The whole model was covered with 8.5 mm Tero ceramic tiles made by Paradyz. The joints between the tiles were filled with C2S1 deformable cement mortar. The experimental model together with the composite, namely a 20 cm × 20 cm section of floor constructed in the same way, which was necessary for placing the passive strain gauges, is shown in Figure 4.

**Figure 5:**
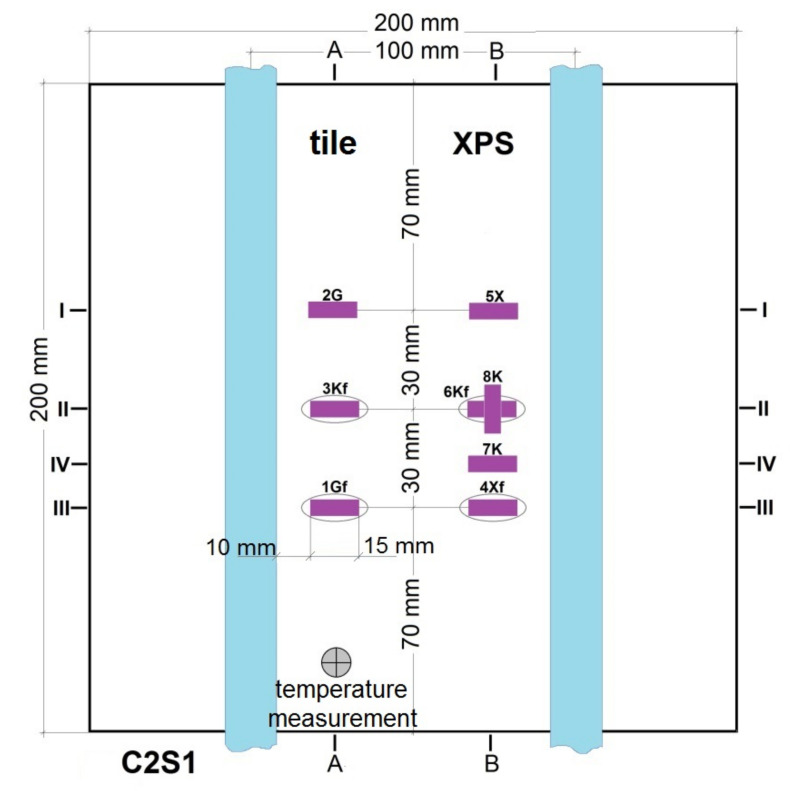
Horizontal section of the test model in the centre with C2S1 adhesive, without lamellas.

**Figure 6:**
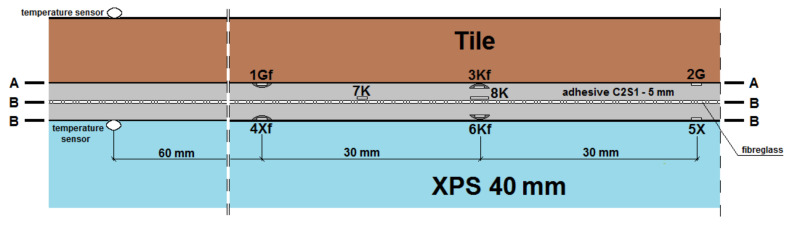
A and B vertical section of the research model with C2S1 adhesive, without lamellas.

**Figure 7:**
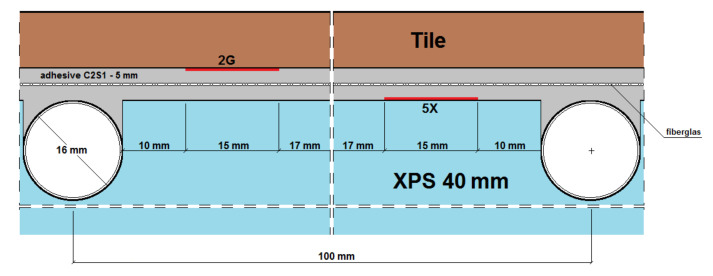
Vertical section I–I of the research model with C2S1 adhesive, without lamellas.

**Figure 8:**
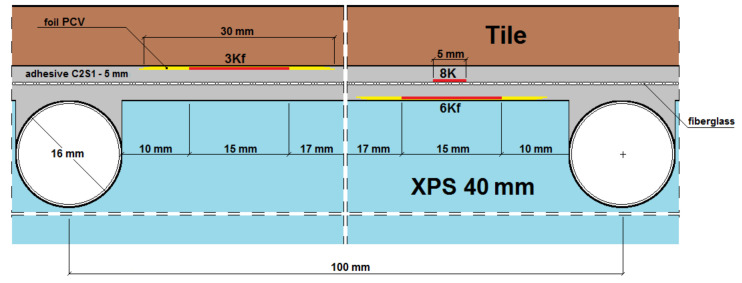
Vertical section II–II of the research model with C2S1 adhesive, without lamellas.

**Figure 9:**
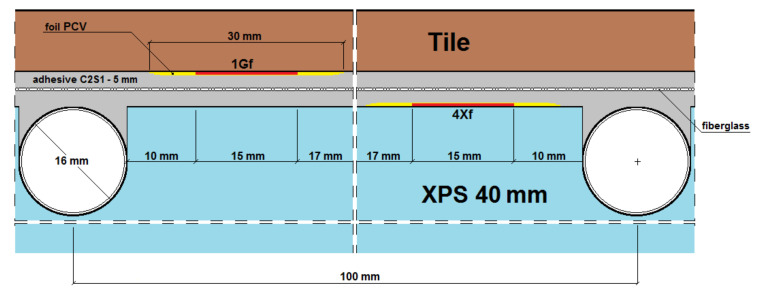
Vertical section III–III of the research model with C2S1 adhesive, without lamellas.

**Figure 10:**
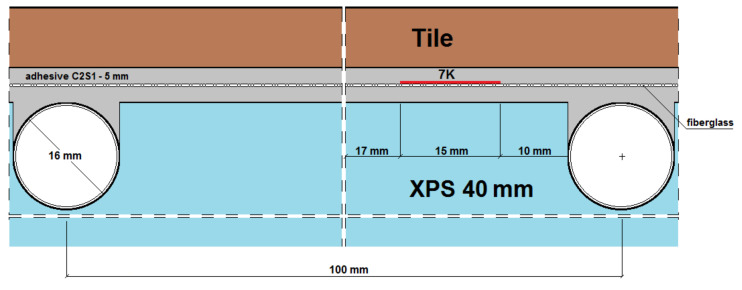
Vertical section IV–IV of the research model with C2S1 adhesive, without lamellas.

Seven strain gauges active transversely to the heating coil were installed on each of the LFS layers. An additional (eighth) strain gauge was attached parallel to the heating pipes to compare the amount of strain in the two mesh directions (along the weft and warp). Deformation was recorded with the use of a set of two 4-channel, 16-bit measuring devices of the SPIDER 8 type by HBM and Catman Express ver. 3.0 release 4 installed on a computer. Strain gauge measurements were performed in the half-bridge system. Temperature compensation was carried out using passive strain gauges placed on an independently prepared, smaller composite of a lightweight floor with dimensions of 20 cm × 20 cm (Figure 4). Tenmex TFs-15 strain gauges were used in the measurements, with a measuring base length of 15 mm, strain gauge constant k = 2.19 ± 0.5%, and resistance R = 120 Ω ± 0.2%. The temperature of the combination of thermal insulation and adhesives was recorded using an electronic thermostat, and on the floor surface using an OJ Electronics TN1 pyrometer (OJ ELECTRONICS, Stenager, Sonderborg, Denmark), with a thermal sensitivity <0.1 °C and a temperature accuracy of +/−2%. The temperature in the centre of the *T_w_* composite was determined using Formula (1) given by Witczak in [21]. The places of installation of active strain gauges and temperature sensors in the central part of the model are shown in Figure 5, Figure 6, Figure 7, Figure 8, Figure 9 and Figure 10.

(1) $$T_{w}=\frac{U_{1}\cdot T_{kl}+U_{2}\cdot T_{po}}{U_{1}+U_{2}}$$

*U*_1_—heat transfer coefficient in adhesives;
*U*_2_—heat transfer coefficient in ceramic tiles;
*T_kl_*—adhesive temperature on the thermal insulation (reading from the sensor);
*T_po_*—temperature on the ceramic tile;
1*G*_f_—strain gauge on the tile, separated from the adhesive by a PVC foil;
2*G*—strain gauge on the tile, covered with glue;
3*K_f_*—strain gauge on the adhesive, separated from the tile by a PVC foil;
4*X_f_*—strain gauge on the XPS, separated from the adhesive by a PVC foil;
5*X*—strain gauge on the XPS, coated with adhesive;
6*K_f_*—strain gauge on the adhesive (from the bottom of the mesh), separated from the XPS by foil;
7*K*—strain gauge on the adhesive, covered with adhesive, placed transversely to the coil (along the weft of the mesh);
8*K*—strain gauge on the adhesive, covered with adhesive, placed along the coil (along the warp of the mesh).

**Figure 11:**
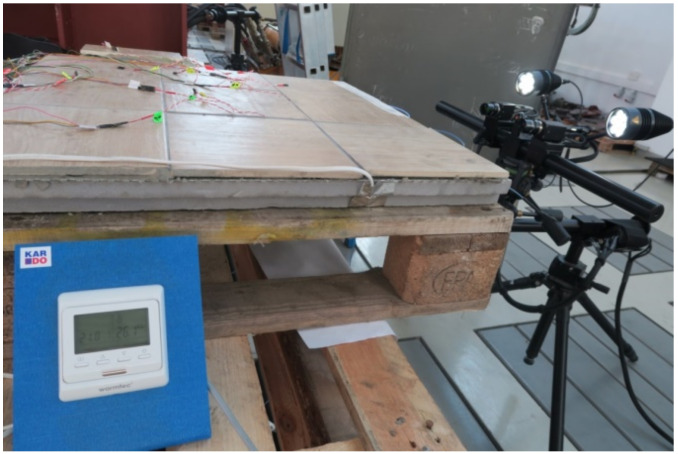
Aramis system test stand for measuring LFS displacements.

In addition to determining the deformations, the displacement of the research model under the influence of temperature changes was checked using the Aramis vision system. The displacements were tested at a maximum width of no more than 240 mm, which resulted from the technical vision capabilities of the DIC system. When examining the image, the DIC system was positioned at one location, measuring displacements, from the edge to points near the centre of the model. The test stand is shown in Figure 11.

**Figure 12:**
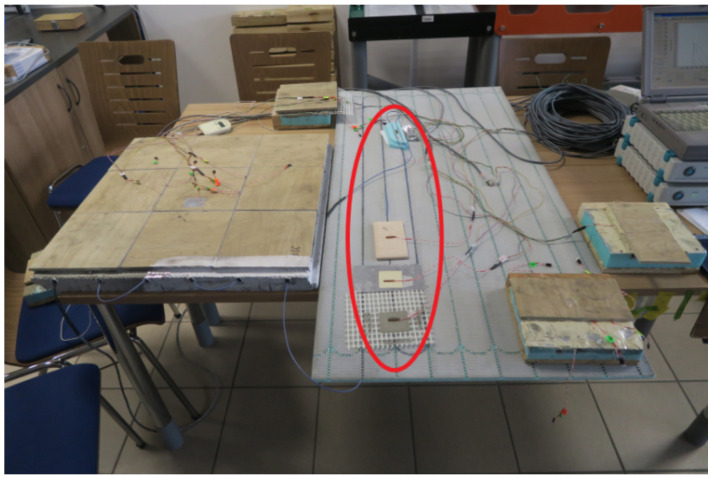
Stand for the correction of component materials in the LFS without lamellas.

The entire study was completed with the measurement correction of the strain gauges placed on the materials used. The correction of the results was a necessary component of the entire process of experimental research under the influence of temperature. The same research apparatus was used for this purpose. Figure 12 shows the materials subjected to correction with strain gauges, marked inside the red ellipse. The rationale for and necessity of making this correction are described in Section 3.

## 3. Results and Discussion of Experimental Methods

The strain gauge measurements, which allow the deformation of the tested materials under the influence of temperature to be determined, consist of several processes. In this type of experiment, it is not sufficient to read the results coming directly from the recorder. These are influenced by temperature, which changes the length of material layers, as well as indications of strain gauges undergoing deformation. The change in material deformation recorded on the strain gauges should be taken into account by correcting the measurement results obtained from the recorder. This reaction of strain gauges to temperature is called “apparent strain” [22,23]. The temperature reaction *Ɛ_v_* is influenced by many factors, most notably the following:

- Thermal expansion of the component material used *α_C_*;
- Thermal expansion of the strain gauge mesh material *α_M_*;
- Temperature electrical resistance coefficient of the strain gauge material *α_R_*;
- Temperature variation d*T* as an inducing variable.

These parameters are listed in Formula (2), with which an approximate calculation of the temperature reaction of the strain gauge *Ɛ_v_* can be made.

*Ɛ_v_* = (*α_R_/k* + *α_C_* − *α_M_*) d*T* (2)

where *Ɛ_v_* is the temperature correction (temperature reaction of the strain gauge), *k* is the strain gauge constant, *α_R_* = 0.49 (Ω × mm^2^/m) or (−60)–(−80) 10^−6^ [1/K], *k* = 2.19, *α_M_* at 20–100 °C = 13.5 × 10^−6^, CuNi44—Konstantan at 20–200 °C + Isotan foil (data from the manufacturer—Tenmex).

Then, according to Formulae (1) and (2), the corrections of the strain gauges used at the reference temperature, e.g., 20 °C, depending on the tested materials, will take the following values:

*Ɛ_v.C_*_2*S*1_ = (*α_R_*/*k* + *α_C_* − *α_M_*) d*T* = (−60/2.19 +12 − 13.5) × 10^−6^ ·d*T* = −29 × 10^−6^
d*T*, (3)

*Ɛ_v.tile_* = (−60/2.19 +8 − 13.5) × 10^−6^ ·d*T* = −33 × 10^−6^
d*T*, (4)

*Ɛ_v.XPS_* = (−60/2.19 +70 − 13.5) × 10^−6^ ·d*T* = +29 × 10^−6^
d*T*. (5)

**Figure 13:**
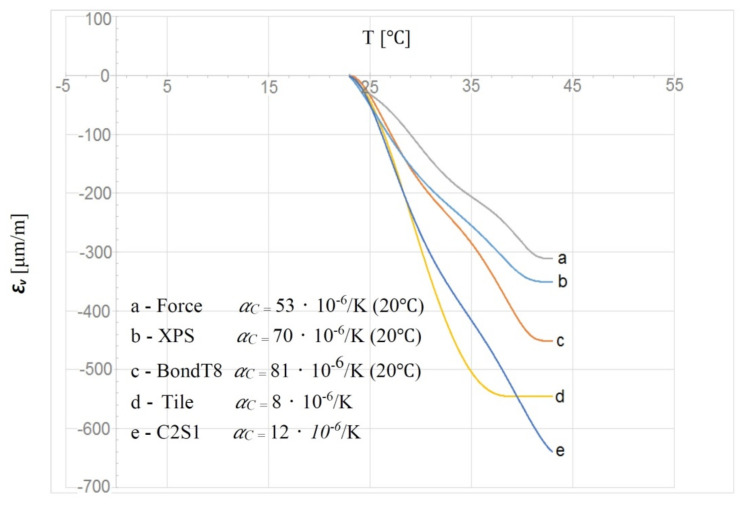
Temperature reactions of strain gauge deformation *Ɛ_v_* mounted on different measuring materials.

The value obtained in this way can be taken as a guiding value for a given temperature range, bearing in mind that *α_C_* may vary with temperature in some materials. This is especially true of flexible materials with low “stiffness”, such as polyurethanes. Hence, the presentation of the temperature reaction (apparent deformation) of various materials, including the components of LFS without lamellas (ceramic tile, C2S1 adhesive, XPS) is more correct in the form of a function, which has been confirmed by Hoffman [22]. If strain gauges with identical parameters are attached to the constituent materials with different *α_C_* values, we will obtain different apparent deformation curves *Ɛ_v_*. Based on experimental studies, preliminary results of material deformation in the glued SLP composite were developed, and then they were corrected by subsequent experimental tests, by mounting the strain gauges on individual materials independently, without gluing the layers. By subtracting from the preliminary experimental results the data of the correction results (Figure 13), the target deformation results for each of the materials used were obtained. In addition to the materials used in the LFS without lamellas, Figure 13 shows the temperature reactions of the BondT8 and Force polyurethane adhesives used in the LFS with lamellas.

It should be mentioned that the measurement, during which the measured object experiences both temperature changes and mechanical load, results in the sum of the mechanical and thermal deformation. Assuming only one of these deformations without taking into account the correction value is a mistake. These measurement errors in the event of thermal influence can also be eliminated by using temperature compensated strain gauges or compensation techniques, as confirmed by Hoffman in [22]. In this experiment, the mechanical load was only due to the dead weight of the LFS.

It was noticed that another way to compensate for the initial results may be to use Formula (6), resulting from the shortening of Formula (2), without taking into account the thermal expansion of the component material *α_C_* used. This method of correction, resulting from the elimination of strain gauge deformation, can be called theoretical.

***Ɛ**_v.teor._* = (*α_R_/k* − *α_M_*) d*T* (6)

With the use of a strain gauge with a constant k = 2.19, the obtained theoretical correction value was equal to:

*Ɛ_v.teor._* = (−60/2.19 − 13.5) × 10^−6^d*T* = −40.9·10^−6^
d*T* (7)

By subtracting the value of the theoretical correction *Ɛ_v.teor._* from the preliminary experimental results, we obtain the value of the deformation (expansion) of a given material.

The theoretical Formulae (2) and (6), as given in [22,23], can only be applied to “rigid” materials. All materials (except ceramic tiles) used in the composite, such as XPS insulation, and even C2S1 deformable cement adhesive, are flexible materials. The mesh used in the experimental model does not significantly increase the stiffness of the composites. A comparison of the deformation value corrections was performed based on the experiments, which is reflected in Figure 13 and the calculations from Formula (6). The final correction of the deformation of the LFS component materials without lamellas was made based on the experimental correction data because they confirmed the results of numerical calculations. The conclusion is that only materials with high “stiffness”, when we want to correct their deformations based on the mentioned formulas, can give measurable results. Adjustments to “flexible” materials should be obtained through experimentation. If we want to be sure that our correction of the results, obtained experimentally or computationally from the formulas, is correct, then when compensating for this type of research, it is worth verifying the obtained data by comparing them to those obtained by computer calculations.

The final results after correcting the deformation results in the materials and interface zones of the lightweight floor are given in Table 1. No deformation corrections were made in places where there was no foil separation, considering the experimental data as unreliable (too high). The reason for the measurement error was faulty readings of strain gauges in places of their double-sided adhesion (with cement adhesive) to other layers of the floor.

**Figure 14:**
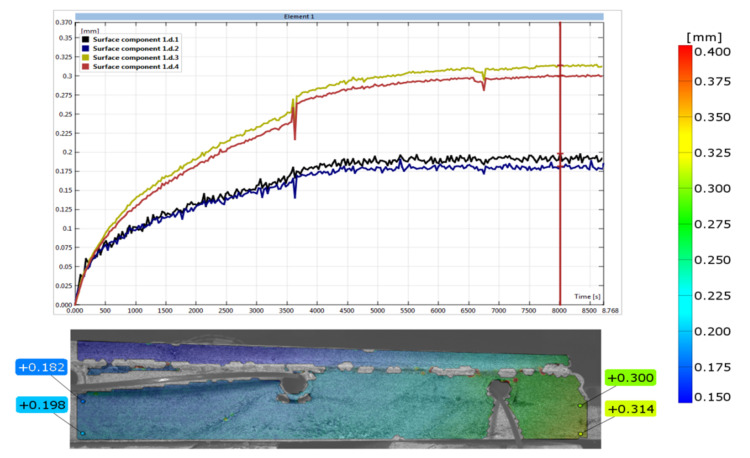
The number of displacements between the centre and the edge of the floor.

The values and manner of displacement were measured using the digital image correlation (DIC) method on a span of 232 mm and shown in Figure 14. The values of displacement on a span of 232 mm was taken 8500 s after the start of the experiment. It was measured at the points shown in Figure 14 and was 0.118 mm on average (0.300–0.182 mm). Data in Table 1 confirm that under the influence of temperature all adopted LFS materials without lamellas are subject to expansion.

**Figure 15:**
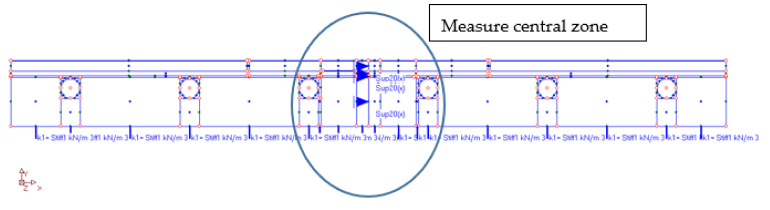
The computer model of the studied structure.

**Figure 16:**
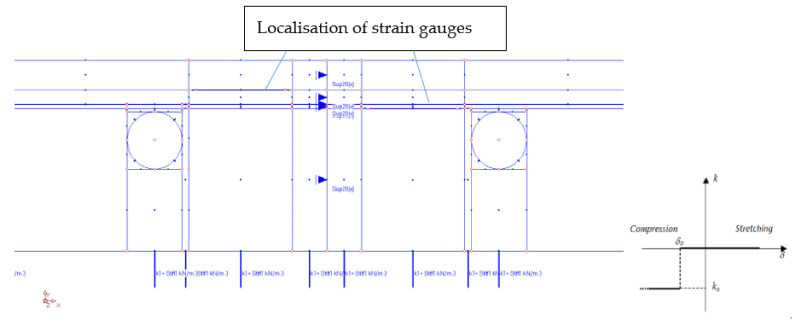
Measurement zone and substrate modelled by “contact zone”. *k*, *k_o_*—spring stiffness; *δ*, *δ*_o_—displacement.

After the completion of the experimental research, numerical verification with the finite element method (FEM) was started. For this purpose, the “ORCAN” structure analysis system was used, which was fully implemented at the Bialystok University of Technology, and the Ansys computer program was used to verify the results. The displacements were measured at the points shown in Figure 14. These displacement readings were taken 8500 s after the start of the experiment. Figure 15 shows the mapping of the experimental model with places of measurement of displacement and deformation on the edges of the central zone. The joints were designed between the tiles. The deformations were measured using strain gauges attached to the surfaces of the floor layers. The arrangement of the strain gauges is shown in Figure 16. For verification of the experiment, the temperature data from Table 1 was used: on the external surface of the tile, 9.5 °C; on the joint between the stoneware adhesive, 10.1 °C; and at the interface of adhesive and XPS insulation, 10.5 °C. The temperatures corresponded to a research time of 8500 s. The installation temperature was according to Table 1, namely *To* = *Ti* = 23 °C. The model was loaded with self-weight only. The research model was placed freely on the substrate. This substrate was modelled with a so-called “contact zone”, which works like a spring. The joints of C2S1 were designed between the tiles. 

**Figure 17:**
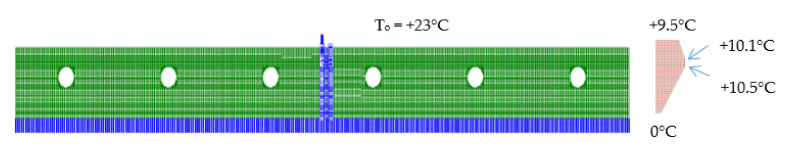
Model discretization with temperature actions in the floor.

**Figure 18:**
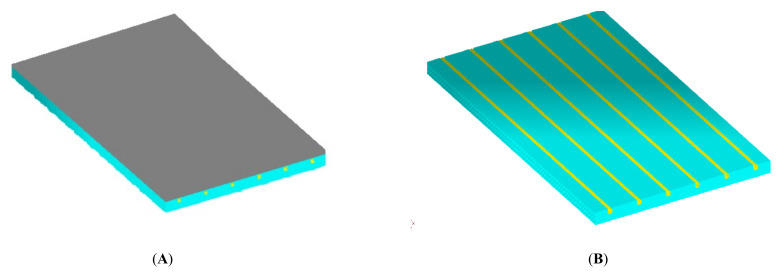
The 3D visualization of the model: (**A**) sample with an adhesive, (**B**) location of heating pipes.

In numerical calculations, flat finite elements, working in a plane stress state (PSS) with a unit thickness of 1.0 m, were used. Contact with the ground was modelled with special one-dimensional spring-type finite elements, in which the change in stiffness was used, depending on the direction of internal forces in the spring. Horizontal support was applied in the axis of symmetry (Figure 16) to stabilize the computational model. The division into finite elements with the temperature actions in the floor is shown in Figure 17, and the 3D visualization of the model in Figure 18.

**Figure 19:**
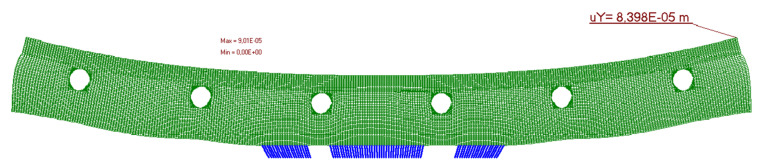
Deformation of the model.

**Figure 20:**
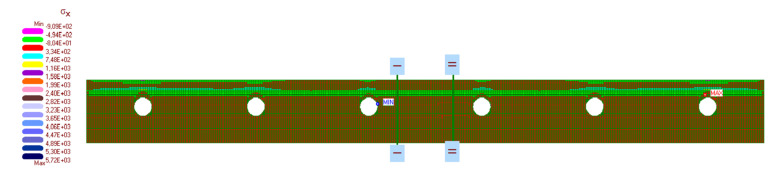
The map of horizontal stresses *σ_x_* and cross-section location.

**Figure 21:**
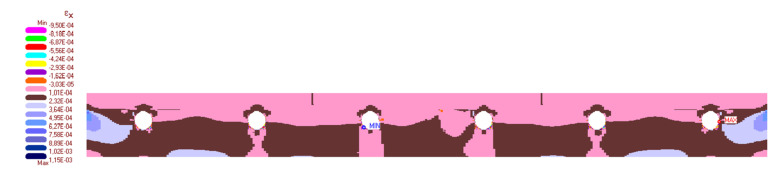
The map of horizontal deformations *ε_x_*.

**Figure 22:**
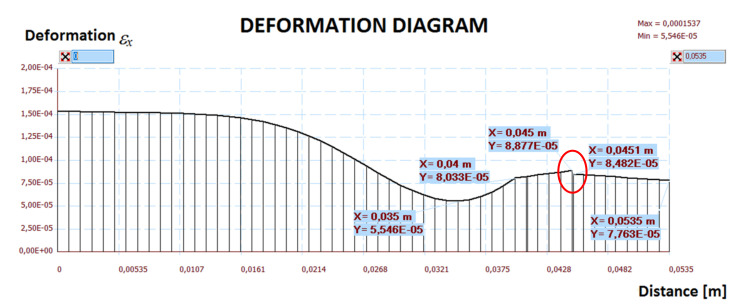
The diagram of horizontal deformations *ε_x_* in the I–I section.

**Figure 23:**
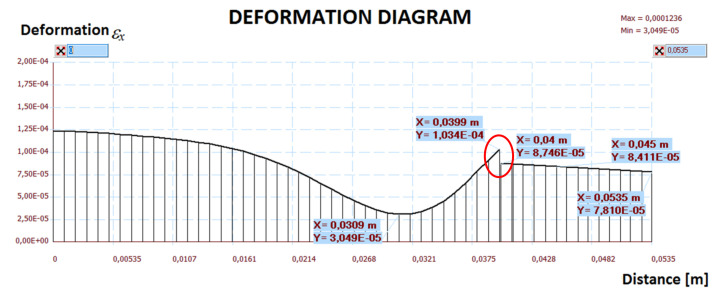
The diagram of horizontal deformations *ε_x_* in the II–II section.

The calculation results are presented in Figure 19, Figure 20, Figure 21, Figure 22 and Figure 23. Figure 19 shows the method of deformation and the amount of model displacement under the influence of the temperatures shown in Figure 17. The read displacement at the edge of the model was 8.4 × 10^−5^ m and could be compared to the displacement from the experiment. Sections I–I and II–II (Figure 20) were drawn through the places where the strain gauges were installed, and the horizontal stresses *σ_x_* were read in them. Figure 21 shows the floor deformation maps from the assumed loads. Figure 22; Figure 23 show graphs of horizontal deformations of the floor layers in two sections, and the deformations occurring at the attachment points of the strain gauges are marked in red. The values of these deformations could be compared to the deformations obtained in the experiment for 8500 s (Table 1).

**Figure 24:**
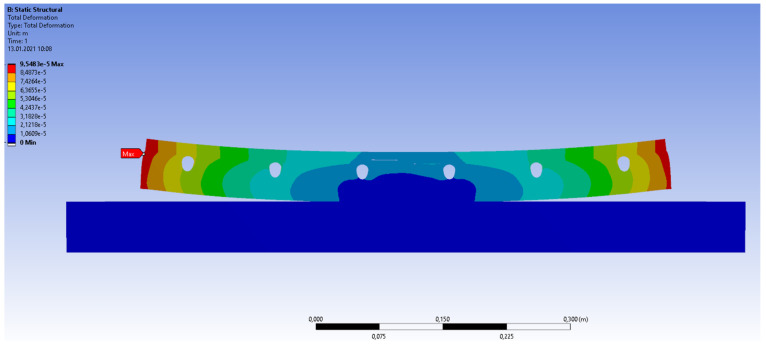
System deformation in the Ansys program.

**Figure 25:**
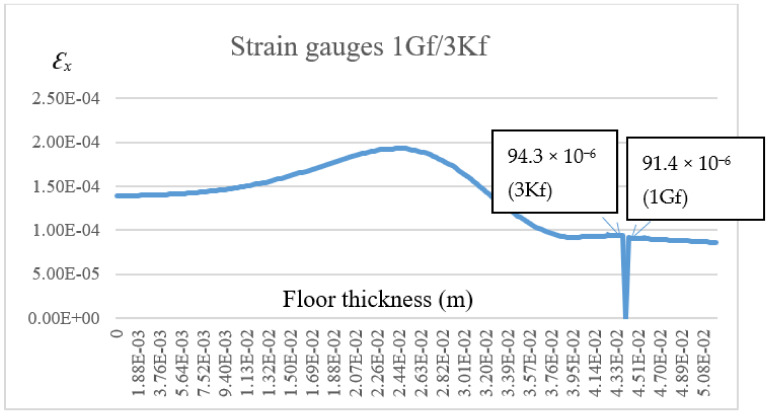
The diagram of horizontal deformations *ε_x_* in the I–I section.

**Figure 26:**
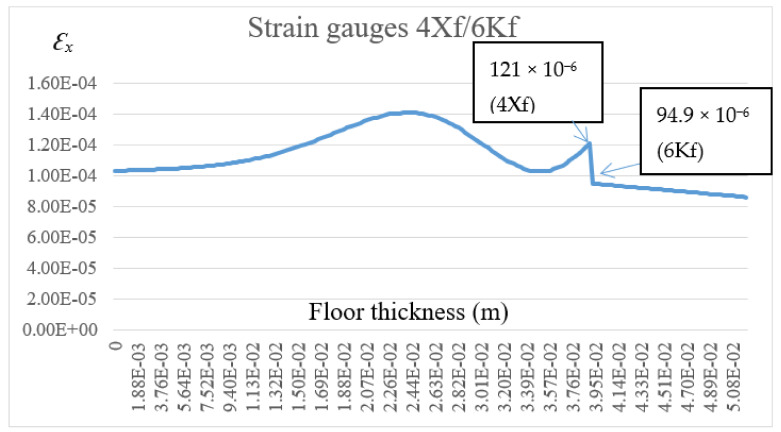
The diagram of horizontal deformations *ε_x_* in the II–II section.

To verify the obtained data through the “Orcan” structure analysis system, a computational model was built using the Ansys program. The floor defection and deformation results are shown in Figure 24, Figure 25 and Figure 26. Figure 25 and Figure 26 show graphs of horizontal deformations of the floor layers in two sections, and the deformations occurring at the attachment points of the strain gauges are marked by the arrows.

A comparative summary of the numerical verification results with the experimental results and comments are presented in Table 2.

The displacement values of the lightweight floor composite obtained in the numerical model were on average 0.084 mm. Relative deviations of the deformations, calculated with two computer programs, were not greater than 12.6% (ORCAN) and 10.3% (ANSYS). The difference between the ORCAN and ANSYS systems resulted from the fact that the ORCAN system simplifies the description of the temperature, taking it as the average for the entire finite element. This is due to the small dimensions of the finite elements. The comparison of the results of experimental and numerical tests showed that they were convergent and sufficiently precise, taking into account the use of materials with very different material parameters (*E*, *ν*, *α*) and different temperatures occurring on each of the layers of a lightweight heated floor.

**Figure 27:**
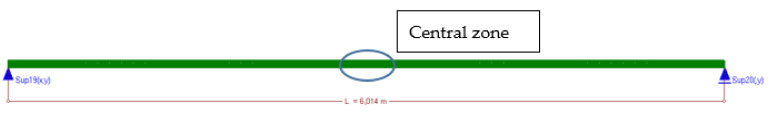
Computer model of the research structure.

**Figure 28:**
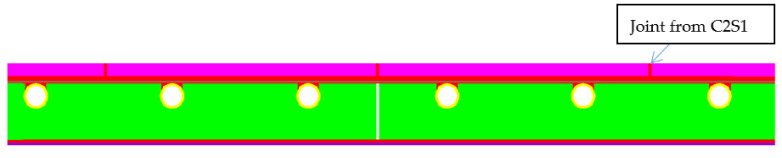
The floor layers—middle zone.

**Figure 29:**
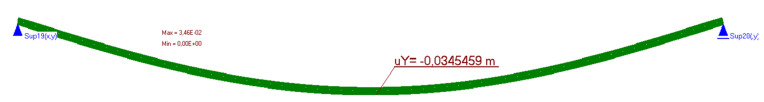
Deformation of the model.

After confirming the compliance of the experimental and computational results on the model with dimensions of 60 cm × 60 cm, numerical verification of the stresses at maximum deflections was started, building a computational model of a lightweight floor with the thermal action, paced on a reinforced concrete ceiling with a span of 6 m (Figure 27). Figure 28 shows the central zone arrangement with all layers of the floor. The maximum deflection was achieved by applying a uniformly distributed load of 35 kN/m^2^ to the reinforced concrete ceiling. The numerical tests aimed to check the stress of the floor structure with cement adhesive, with the maximum standard deflection given in the European standard [24], corresponding to 1/200 of the ceiling span. To verify this, the following assumptions were made:

- The standard deflection corresponded to 1/200 of the ceiling span (assumed to be 34 mm; Figure 29);
- The lightweight floor model with C2S1 adhesive was repeatedly duplicated up to a ceiling span of 6.00 m;
- The XPS insulation substrate was glued to the ceiling with cement glue;
- The reinforced concrete ceiling had a thickness of 20 cm;
- The joints were adopted as in the experimental model, and gaps between the XPS panels were also made;
- The adhesive and mesh separation model was used, and the mesh was modelled separately in PSS;
- The self-weight, the imposed load of 2 kN/m^2^, and the thermal action were all the same as in the experimental model.

**Figure 30:**
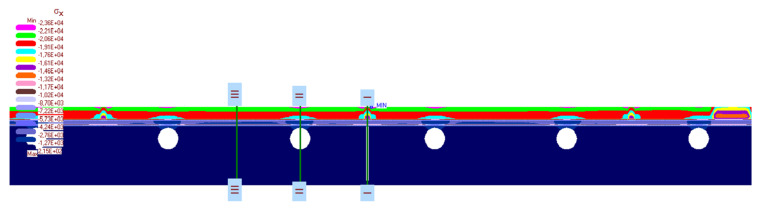
Map of horizontal stresses *σ_x_* in the middle zone and cross-sections location.

**Figure 31:**
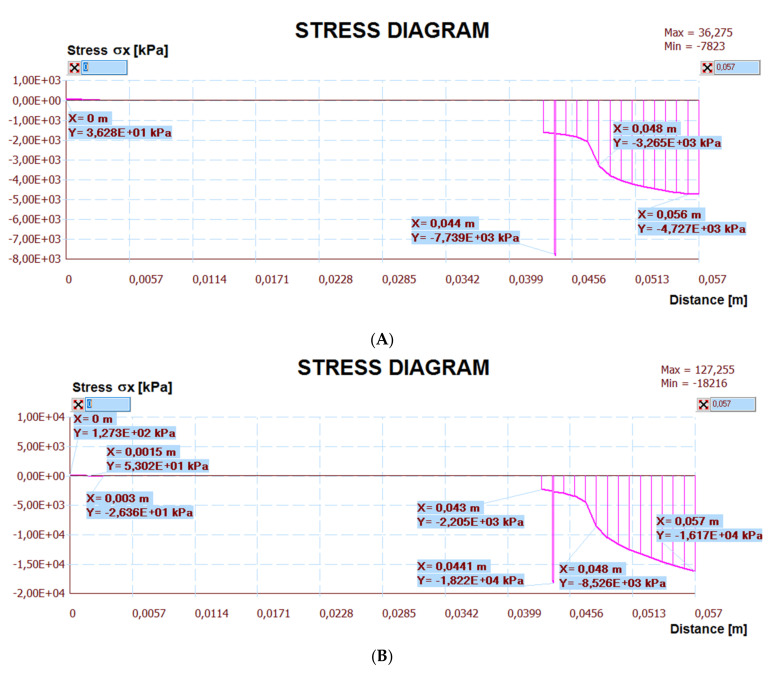
Diagram of horizontal stresses *σ_x_* in the I–I section: (**A**) thermal action without ceiling deflection; (**B**) thermal action and imposed load with ceiling deflection.

**Figure 32:**
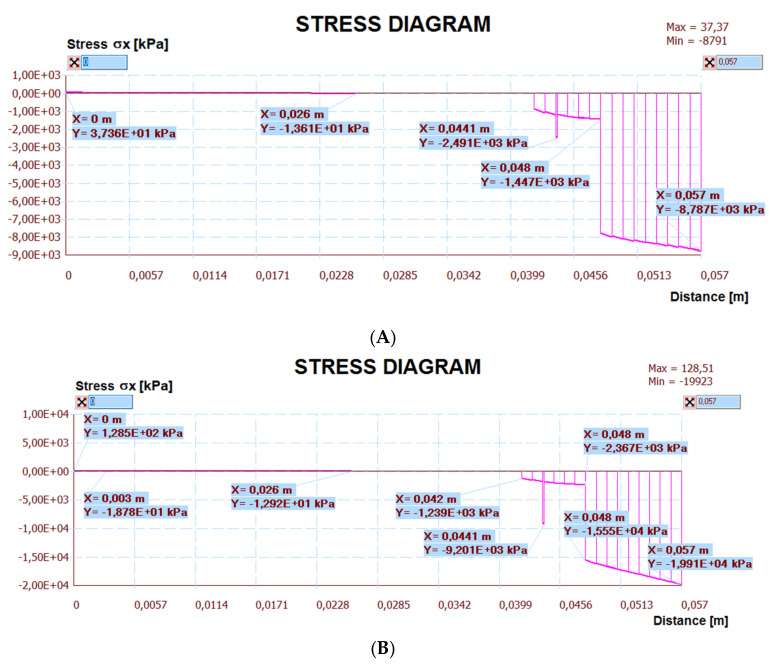
Diagram of horizontal stresses *σ_x_* in the II–II section: (**A**) thermal action without ceiling deflection; (**B**) thermal action and imposed load with ceiling deflection.

**Figure 33:**
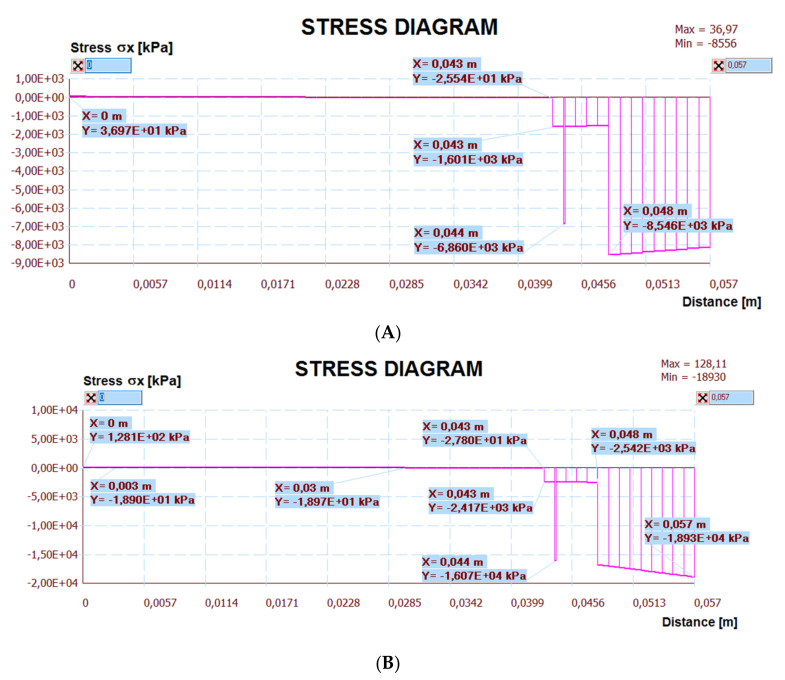
Diagram of horizontal stresses *σ_x_* in the III–III section: (**A**) thermal action without ceiling deflection; (**B**) thermal action and imposed load with ceiling deflection.

The calculation results are presented below. Figure 30 shows a map of the horizontal stresses of the middle zone along with the location of three sections, including I–I located at the break in the thermal insulation connection (centre of the calculation model), which also passed through the tile joint. Cross-section II–II runs through the heating pipe, and cross-section III–III in the place of individual layers of the lightweight floor without lamellas. Figure 31, Figure 32 and Figure 33 show diagrams with the results of horizontal stresses in three sections, with the division of results into the following:

- Thermal interaction with the floor self-weight, without the maximum standard deflection of the ceiling;
- Thermal action with the floor self-weight and imposed load together with the maximum standard deflection of the ceiling.

To numerically verify the deflections, apart from the horizontal stress map, vertical and tangential stresses in places where these stresses are extreme were also analysed. The extreme results of the lightweight floor are presented in Table 3, where the stresses in selected cross-sections corresponding to the horizontal, vertical, and tangential strength are compared with comments. The values of the stress are shown in the variant T, the thermal action without ceiling deflection, and T + U, the thermal action and imposed load with the maximum deflection of the ceiling, separated by the “/” sign. Other sections included in Table 3, indicated in brackets as IV or V, were chosen after analysing the stress maps in other load simulations, e.g., at maximum deflection of the floor and thermal action, but without taking into account the imposed loads. As a result, additional data of the maximum stress were obtained. Analysing the map of shear stresses, it was noticed that the highest shear stresses occurred at the edges of the floor, which was confirmed by the analytical methods for determining the stresses of glued joints described by da Silva et al. in [25,26].

As seen in Table 3, the stress of joints between tiles modelled with C2S1 adhesive, when the load T + U was assumed, was greater than its strength (15.3 MPa strength <16.17 MPa stress). It should be taken into account that the adopted maximum deflection value was overestimated, as the ceiling was already pre-deflected before its construction. The numerical calculations consciously did not take into account the aspect of the initial deflection caused by the weight of the structure itself before the floor layers were made, assuming the possibility of extreme conditions of use of the reinforced concrete floor structure. However, the lack of maximum standard deflections and imposed loads of 2 kN/m^2^ (there are only thermal actions and the floor’s own weight, which can often occur during the use of the building) results in large reserves of load capacity in each of the LFS layers, e.g., in the joint between the tiles 4.73 MPa < 15.3 MPa (Table 3). Generally, the calculated stresses of the LFS layers were significantly lower than the strength of the materials used.

Other calculation simulations (e.g., without taking into account the imposed loads but with maximum defections—which are not in Table 3) confirmed that the condition of the load capacity of the floor layers and its adhesive connection with the ceiling were met:

- The maximum compressive stress in the tiles was 25.5 < 240 MPa (tiles strength) and in the C2S1 adhesive was 9.87 < 15.3 MPa;
- The maximum vertical stress ***σ_y_*** in the C2S1 adhesive, which connected the ceiling to the XPS thermal insulation, was 0.007 < 0.14 MPa (***σ_ymax_***—detachment strength).

## 4. Conclusions

The article presents a lightweight floor system (LFS) with an original layer arrangement developed by the author. This system is mainly studied in the field of environmental engineering, in which research shows many advantages of a lightweight system, especially high efficiency and comfort of use. In this article, an LFS in the mechanical strength field was tested. For this purpose, the correctness of the adopted concept was verified by performing experimental tests, which were verified numerically with the use of FEM. The analysis of the obtained results allows for the formulation of the following conclusions:

1. It was found that the measured values of deformation at the boundaries of the LFS system layers do not differ significantly from the results obtained from the numerical model. The differences amount to a maximum of 12.6% (see Table 2). In addition, the measured displacements at selected points of the model are consistent. Therefore, the correctness of the computational model and the correctness of the experimental research were confirmed. The computational model was simplified to the plane stress state (PSS). This simplification allowed for the use of less computing power while maintaining the appropriate accuracy.
2. The concept of the computational model was adopted as a plane model with the use of two-dimensional finite elements working in the plane stress state (PSS). This was explained by the negligible friction between the floor composite and the substrate, which is related to the low weight of the lightweight floor and the lack of the imposed load. The test sample was lying freely on the substrate. This type of model solution is also indicated by the actual boundary conditions of the floor, where a expansion space is left between the floor and the walls, constituting the room boundaries, in order to possible thermal deformation. Hence, the adopted model is a justified simplification of the three-dimensional model (3D model).
3. Based on the measured deformations, the internal forces in the form of stresses were calculated. These values were consistent with the calculation model, which confirmed the correctness of the adopted concept of converting strains into stresses. In the working conditions of the floor (i.e., with working loads—the temperature of the adhesive layer up to 35 °C and the self-weight), the strength of the LFS components was not exceeded.
4. The computational model was also used for the computer simulation of the floor structure response under extreme operating conditions. The maximum allowable standard deflection of the ceiling was assumed to be l/200, and the floor to the ceiling was joined with type C2S1 adhesive. The results of the analysis showed that in the model without lamellas, the stresses in the cement joints in the middle of the floor may be exceeded, and their chipping may occur. It should be noted that the adopted maximum deflection value is overestimated, as the ceiling is already pre-bent before the floor is built in. The numerical calculations consciously did not take into account the aspect of the initial deflection caused by the self-weight of the structure before the floor layers were made.
5. Based on the performed tests and analyses, it can be concluded that the presented LFS lightweight floor system without screeds can be safely used.

Taking into account the presented results and analyses of the different loads, including thermal actions, it was found that the created model of a lightweight floor system without a screed layer, with a heating coil, meets the strength and deformation conditions under the assumed operating parameters. These positive results can be used in the design and strength and deformation analyses of other types of lightweight heating floors (for example with lamellas) and various building partitions. Thanks to this work, scientists have the opportunity to compare its results with their own research conducted in this or a similar field. Particular attention should be paid to tests carried out under the influence of temperature with the use of strain gauges, bearing in mind the need to correct the obtained preliminary results, which is emphasised in the article. The correction of the data is more accurate when we use research results rather than formulas. In addition, if we want to obtain reliable test results using the strain gauge technique, reliable results are obtained when the strain gauges fixed in the composite are separated, e.g., with foil, from particular layers of this composite.

## Figures and Tables

**Table 1:** Data of material deformation taking the corrections of strain gauges with C2S1 adhesive (average air temperature *T_i_* = 23 °C, max adhesive temperature on the sensor *T_kl_* = 35 °C).

Adhesive /*T_kl_/T_w_*		*T_po_*	(*T_po_ − T_i_*)	(*T_w_ − T_i_*)	(*T_kl_ − T_i_*)	Research Time	Deformation after Correction (μm/m)			
	(°C)	(°C)	(°C)	(°C)	(°C)	(s)	1*G_f_*	3*K_fg_*	4*X_f_*	6*K_fx_*
C2S1	24/23.8	23.5	0.5	0.8	1	500	10	5	31	8
	25/24.8	24.7	1.7	1.8	2	700	21	14	54	20
	26/25.8	25.5	2.5	2.8	3	1200	23	17	80	25
	27/26.8	26.7	3.7	3.8	4	1500	30	34	87	40
	28/27.8	27.6	4.6	4.8	5	2250	39	37	94	46
	29/28.7	28.3	5.3	5.7	6	3200	47	44	96	58
	31/30.6	30.0	7.0	7.6	8	5500	54	59	114	70
	33/32.6	32.0	9.0	9.6	10	7000	95	83	115	97
	34/33.6	33.0	10.0	10.6	11	10,000	99	88	119	102
	34.5/34.1	33.6	10.6	11.1	11.5	11,500	107	93	124	116
	35/34.7	34.2	11.2	11.7	12	22,000	80	75	135	136
	35/34.7	34.3	11.3	11.7	12	38,250	73	30	140	141

**Table 2:** The list of model deformations.

Measurement Point	Computational Deformation O/A	Measured Deformation	Relative Deviation (%)	Comments
1Gf	+84.8 × 10^−6^/+91.4 × 10^−6^	+97.0 × 10^−6^	12.6/ 5.8	Strain gauge glued to the tile, separated from the adhesive
3Kf	+88.8 × 10^−6^/+94.3 × 10^−6^	+85.5 × 10^−6^	3.8/ 10.3	Strain gauge glued to the adhesive, separated from the tile
4Xf	+103.4 × 10^−6^/+121.0 × 10^−6^	+117.0 × 10^−6^	11.6/ 3.4	Strain gauge glued to the XPS, separated from the adhesive
6Kf	+87.5 × 10^−6^/+94.9 × 10^−6^	+99.5 × 10^−6^	12.1/ 4.6	Strain gauge glued to the adhesive, separated from the XPS

O—calculations using the “Orcan” program; A—calculations using the “Ansys” program.

**Table 3:** The stresses in the particular materials of the lightweight floor.

Layer	Stress in Sections (T/T + U) (MPa)		Strength (MPa)	Comments
	σ_x_	σ_y/_//τ		
Tile/ C2S1 (I)	⊖ 4.73/16.17 (I) ⊖ 8.79/19.91 (II)	⊖ 1.48/2.76 (I) ⊖ 4.79/6.12 (III/V) //0.12/0.88 (V)	⊖ 15.3 ⊖ 240 ⊕52.0	Large stocks of load capacity in ceramic tile. Exceeding the strength of the adhesive in the joint, at *σ_x_* (T + U)
C2S1	⊖3.27/8.53 (I)	⊖ 1.12/1.46 (II/I) //0.05/0.25 (V)	⊖ 15.3 ⊕ 0.14 - *σ_ymax_*//0.4	Large stocks of load capacity in adhesive
GFRP (fiberglass mesh)	⊖ 8.99/18.08 (V) ⊕ 2.72/⊖ 9.2 (II)	-	⊖ (-) ⊕ 1350	Large stocks of load capacity in the mesh. No compression failure, marked by (-)
XPS	⊖ 0.026/ 0.028 (V)	⊖ 0.017/0.021 (V/IV) //0.003/0.013 (IV)	⊖ 0.30 ⊕ 0.40//0.15	Large stocks of load capacity in XPS
C2S1	⊕ 0.037/0.13 (I–V)	⊖ 0.005/0.013 (I) //0.004/0.024 (III)	⊖ 15.3 ⊕ 1.35 //0.4	Large stocks of load capacity in connection with the ceiling

⊕—tensile stresses, ⊖—compressive stresses, ***σ_ymax_***—max. detachment stresses, T—thermal action without deflection of the ceiling, T + U—thermal action and imposed load with the maximum deflection of the ceiling.

## Data Availability

The data presented in this study are available upon request from the corresponding author.
